# Football Size Jejunal Gastrointestinal Stromal Tumour

**DOI:** 10.7759/cureus.11913

**Published:** 2020-12-05

**Authors:** Sherif Monib, Hany F Habashy

**Affiliations:** 1 Breast Surgery, West Hertfordshire Hospitals NHS Trust, St. Albans and Watford General Hospitals, London, GBR; 2 Surgical Oncology, Fayoum University, Fayoum, EGY

**Keywords:** gastrointestinal stromal tumour, gist, abdominal mass

## Abstract

Gastrointestinal stromal tumours (GISTs) are considered the most common mesenchymal neoplasms of the alimentary tract, yet they account for only 0.2% of all gastrointestinal neoplasms.

We are presenting a case of a 68-year-old gentleman who was diagnosed with a 250 mm jejunal GIST only when he presented with abdominal pain and fullness in the upper abdomen. We believe that detailed medical history, followed by prompt investigations, will help in early diagnosis of small GISTs with less malignant potential, which in turn will lead to better outcomes.

## Introduction

Mazur and Clark were the first to describe gastrointestinal stromal tumours (GISTs) in 1983 as rare mesenchymal tumours of the alimentary tract [1]. The annual incidence of GISTs is between one to two per 100,000, with slight male predominance, and a peak of diagnosis around the age of 60 [2]. Although GISTs are the most common mesenchymal neoplasms of the alimentary tract yet they account only for 0.2% of all gastrointestinal neoplasms and only 0.04% of small intestinal tumours; jejunal GISTs are considered the rarest subtype, with only 10-30% malignant potential [3,4].

In spite of the fact that most GISTs present with acute or chronic gastrointestinal bleeding, or unexplained anaemia, we are presenting a case of a gentleman who was only diagnosed with huge jejunal GIST when he presented with abdominal pain and fullness.

## Case presentation

We are presenting a case of a 68-year-old man who presented to the local accident and emergency department with abdominal pain and distension. His past medical history included hypertension and recent intermittent dull aching abdominal pain as well as fullness in the upper abdomen of about one-year duration but he was a bit apprehensive to seek medical advice due to the current pandemic restrictions, he had no history of previous acute illness or hospitalization, and his family history was irrelevant. General examination was unremarkable, and his vital signs were within the normal range. Abdominal examination revealed a tender, palpable mass in the epigastric region, which is even visible with inspection when the patient lies flat.

**Figure 1:**
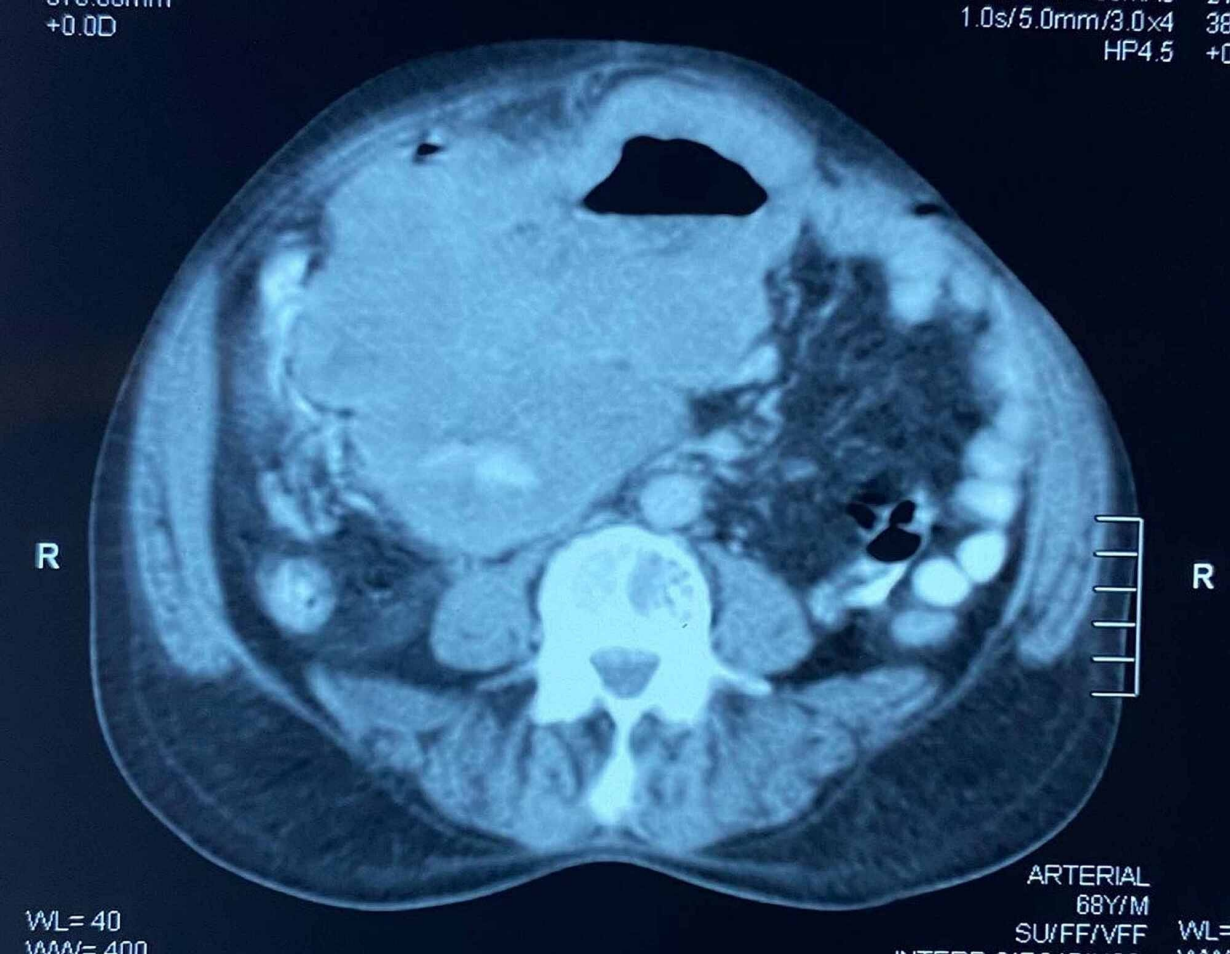
CT scan of the abdomen showing a large gastrointestinal stromal tumour.

**Figure 2:**
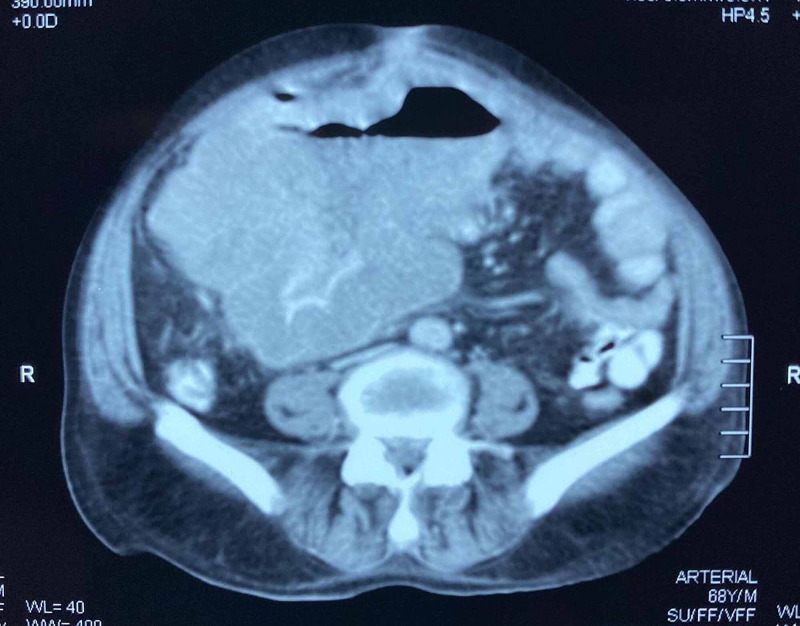
CT scan of the upper pelvis showing a large gastrointestinal stromal tumour.

His laboratory investigations were normal; given the palpable mass, a CT scan of the abdomen and pelvis was carried out which showed a large tumour arising from the proximal jejunum with no signs of obstruction (Figures 1, 2).

**Figure 3:**
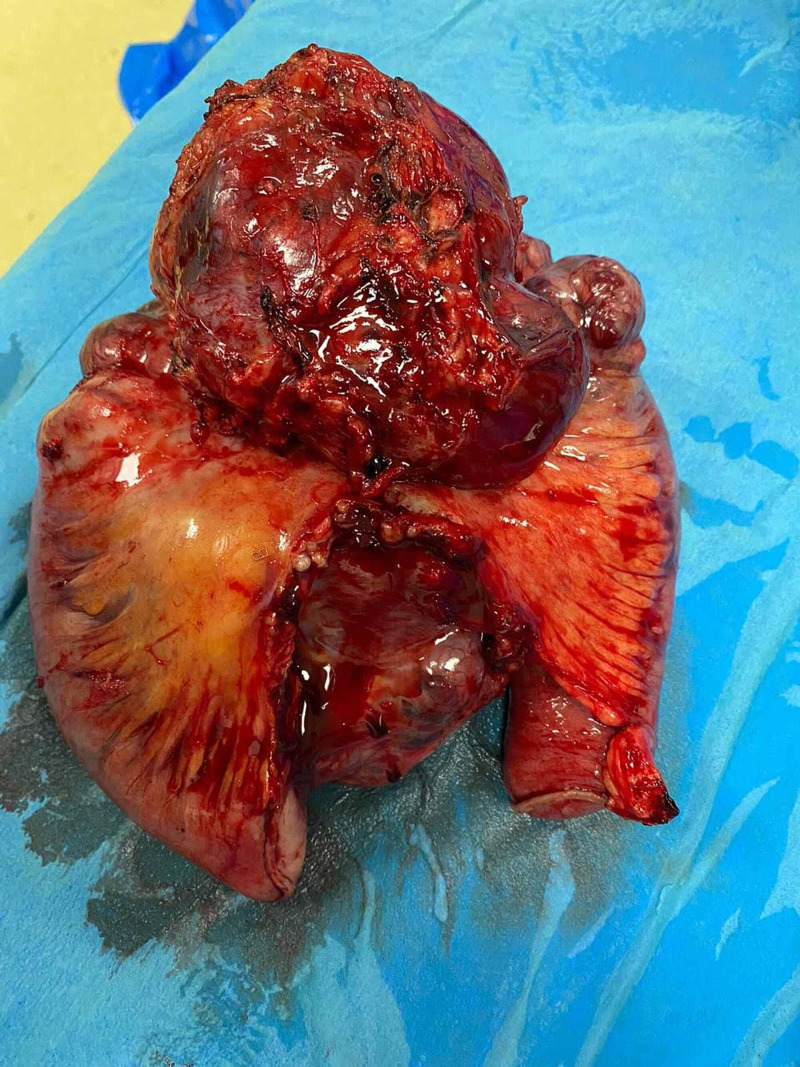
Surgical specimen showing large gastrointestinal stromal tumour with adequate resection margins.

In view of CT scan findings, a planned elective laparotomy was carried out during the same admission revealing a 250 mm GIST at the proximal jejunum about 200mm from the duodenojejunal junction, with no signs of mesenteric lymph nodes or omental or peritoneal nodules (Figure 3).

**Figure 4:**
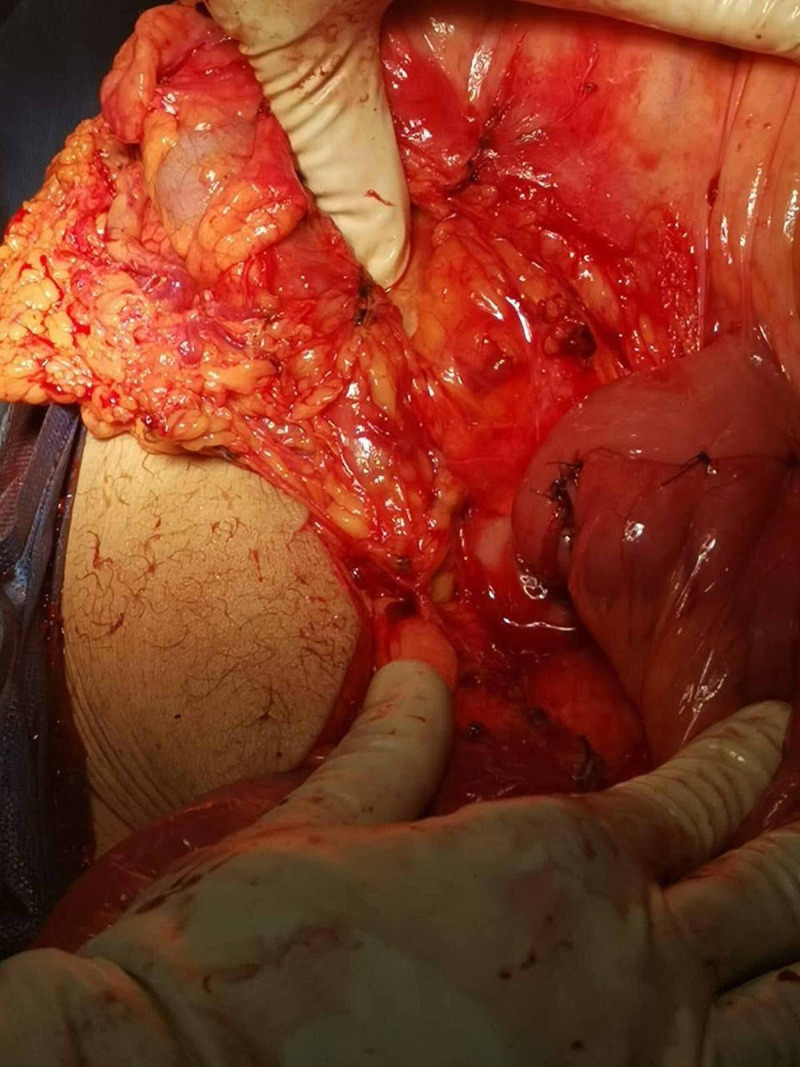
Intraoperative picture showing the primary anastomosis site.

Resection followed by primary stapled side to side anastomosis was carried out (Figure 4).

The patient had an uneventful recovery and was discharged on the fifth postoperative day, seen in the clinic three weeks after showing no signs of postoperative complications.

Postoperative histology confirmed the mass to be a fully resected GIST, with mitotic count > 5/50 high power field (HPF); based on the size and the high mitotic count, imatinib was prescribed to the patient.

## Discussion

GISTs originate from interstitial cells of Cajal (ICC), a are part of the autonomic nervous system of the intestine controlling motility; about 70% of GISTs are found in the stomach, 20% in the small intestine and 10% in the oesophagus. While small tumours tend to be benign, larger tumours have got malignant potential which might lead to liver, omental and peritoneal metastasis [5]. 

Patients with GISTs usually are either asymptomatic or present with nonspecific symptoms as bloating, abdominal pain, and rarely melaena, palpable mass or obstruction secondary to intussusceptions; CT scan is considered the preferred imaging modality to confirm the diagnosis, with radiologic features as; size >50 mm, heterogeneous enhancement, and ulcerations suggesting malignancy [3,6].

As with large GISTs there is always a risk of compression leading to possible bowel obstruction, the current recommendations for assessing the risk of compression rely on tumour location, size, and mitotic index [7]. Also, GISTs are considered to be high-risk with malignant potential and increased risk of recurrence if the tumour is >100mm with any mitotic index, >50 mm with a mitotic count >5/50 HPF, or with ruptured tumours [8]. In our case, the tumour was considered as high-risk as it was 250 mm in maximal diameter, located in the proximal jejunum, with high mitotic count.

Complete resection of high-risk GIST results in 95% five-year survival. For GISTs larger than 10 mm, adjuvant imatinib provides a 14% absolute reduction in recurrence rate and also achieve 97% recurrence-free survival [5]. In view of our patient's GIST size and histological findings, imatinib was prescribed for the patient.

This case is remarkable as we believe it was one of the largest high-risk jejunal GIST presenting without obstructive symptoms recorded in the literature.

## Conclusions

Good medical history, followed by prompt investigations, will help in early diagnosis of small gastrointestinal stromal tumours with less malignant potential, which in turn will lead to a better outcome. We also recommend publishing large jejunal gastrointestinal stromal tumours to be able to come up with updated management evidence-based guidelines.
